# Antihypertensive treatment and blood pressure trends among South African adults: A repeated cross-sectional analysis of a population panel survey

**DOI:** 10.1371/journal.pone.0200606

**Published:** 2018-08-01

**Authors:** Annibale Cois, Rodney Ehrlich

**Affiliations:** 1 Division of Epidemiology and Biostatistics, School of Public Health and Family Medicine, University of Cape Town, Cape Town, Western Cape, South Africa; 2 Division of Occupational Medicine, School of Public Health and Family Medicine, University of Cape Town, Cape Town, Western Cape, South Africa; CUNY, UNITED STATES

## Abstract

**Background:**

Recent findings suggest a decline in mean blood pressure and prevalence of uncontrolled hypertension in the South African adult population in the last decade, in contrast with the increase previously observed. This study aimed at quantifying the contribution of antihypertensive treatment to this decline.

**Methods:**

We used data from the four waves of the National Income Dynamics Study between 2008 and 2015 and analysed changes in systolic (SBP) and diastolic blood pressure (DBP) and prevalence of uncontrolled hypertension among South African adults (15 years and above). We compared the observed changes with a counterfactual scenario in which the impact of antihypertensive treatment was estimated by censored regression and removed, with and without adjustment for BMI, waist circumference, alcohol use and smoking.

**Results:**

During the study period, the prevalence of antihypertensive treatment rose from 13.1% to 17.6% among women and from 5.3% to 8.2% among men. Concurrently–despite worsening trends in major biobehavioural risk factors for elevated blood pressure–mean SBP decreased in both genders, DBP decreased among women and was stable among men. The odds of uncontrolled hypertension decreased by 4%/year among women and 1%/year among men. After removing the treatment effect, the downward trend in the odds of uncontrolled hypertension was reduced to 1%/year among women and completely offset among men. Among those 55 years and older, but not among younger subjects, treatment effects also explained most of the observed decreases in mean SBP and DBP.

**Conclusions:**

Among South African adults, we infer that diffusion of antihypertensive treatment contributed substantially to the downward trend in the prevalence of uncontrolled hypertension observed between 2008 and 2015. The marked decrease in SBP and uncontrolled hypertension found among younger participants could not be explained by treatment nor by the changing distribution of the measured risk factors available in this study, and requires further investigation.

## Introduction

Convincing epidemiological evidence indicates that in the last decades the burden of disease related to elevated blood pressure (BP) has progressively shifted from high-income countries, where population averages of both systolic (SBP) and diastolic blood pressure (DBP) and prevalence of hypertension have been constantly decreasing, to low- and middle-income countries (LMICs). The latter have experienced the opposite trend and are currently bearing the greatest share of the burden.[1–3] In 2010, the age-standardised proportion of adults aged 20 years and over classified as hypertensive according to the common epidemiological definition ─ SBP≥140 mm Hg and/or DBP≥90 mm Hg and/or being on antihypertensive treatment ─ was estimated at 28.5% (95%CI: 27.3% -29.7%) in high-income countries. This represented a decrease by 2.6 percentage points since 2000. The equivalent figure in LMICs was 31.1% (95%CI: 30.2%-32.9%), representing an increase by 7.7 percentage points over the same period. In absolute numbers, this corresponds to 1.04 billion hypertensive individuals living in LMICs, compared to 349 million in high-income countries.[4]

The constant increase of the average BP in most LMICs–only partly explained by the ageing of the population–is usually attributed to their rapid economic development and urbanization with consequent adoption of ‘westernised’ lifestyles characterised by low levels of physical exercise and high consumption of energy-dense, salt-rich processed food.[5] The cumulative effects of inter-generational changes in food availability is also a likely contributing factor, through mechanisms of genetic programming that have also been advanced to explain other changes in health profiles.[6] There is evidence, for example, that relates early-life undernutrition and rapid compensatory growth in children previously undernourished to higher BP and risk of hypertension during adolescence and adulthood.[7]

On the contrary, the decrease in BP in high-income countries has happened despite unfavourable trends in some of its main proximal determinants, chiefly overweight and obesity. This paradox has been mainly (although far from totally) explained by the diffusion of hypertension prevention efforts and programmes, such as salt reduction in processed food and promotion of the consumption of fresh fruits and vegetables. There have also been an increasing awareness of the condition and the application of detailed clinical guidelines for the effective pharmacologic control of blood pressure in hypertensive individuals.[4,8]

A recent large meta-analysis of population studies including data collected between 1975 and 2015 in 200 countries has substantially confirmed the previous evidence regarding contrasting trends between high-income and LMICs.[3] However, it has also shown that the overall increasing trend of BP in LMICs as a whole is the averaged result of very different situations. In particular, despite the ageing of the population and in countertendency to the rapid diffusion of other lifestyle-related diseases, some middle-income regions are currently experiencing clearly *decreasing* trends, similar to those prevalent in HICs.[3][9] This is the case of South Africa ─ a middle-income country in sub-Saharan Africa in full demographic and epidemiological transition[9] ─ where the comparison of a series of cross-sectional estimates carried out in the general adult population in the period 2007–2015 clearly suggest a decreasing BP trend, in contrast with the remarkable increase observed in the previous decade.[10] The non-artefactual nature of this observation is also supported by the observed reversal, after 2000–2005, of the previously increasing trend in mortality and number of disability adjusted life years lost per stroke.[1,11] Elevated BP is, in fact, the most important determinant of the risk of stroke with a linear relationship beginning at relatively low levels of SBP and DBP. A common finding of population studies is that a decline in BP in the population is quite closely followed, with a short time lag, by a reduction in mortality rates for stroke.[8,12]

The reasons of the decreasing BP in the South African population are unclear. In particular, it is unclear which proportion of this decline is attributable to the observed increased diffusion of pharmacological treatment among hypertensive subjects,[13] rather than to changes in population-wide behaviours and environmental conditions. In resource-constrained health systems–such as the South African public sector, which is facing the growing prevalence of non-communicable diseases simultaneously with the persistent burden of infectious diseases [14]–understanding the effectiveness of providing access to antihypertensive drugs to all population strata is crucial for public health policy and planning.

This study aimed at narrowing this knowledge gap and had three objectives. The first was to estimate age-specific trends in mean SBP and DBP in the South African adult population between 2008 and 2015, the prevalence of uncontrolled hypertension and the number of subjects affected. Uncontrolled hypertension was defined as per common epidemiological practice and in accordance with the universal BP treatment targets recommended by the South African hypertension practice guideline[15] as SBP ≥140 or DBP ≥90 mm Hg regardless of antihypertensive treatment. The second objective was to quantify the contribution of antihypertensive treatment to explaining those trends. The third was to compare this contribution with the contribution attributable to changes in the distribution of some known major determinants of BP, namely age, body mass index (BMI), waist circumference, alcohol use and smoking.

## Methods

This study is a repeated cross-sectional analysis of a population panel survey.

### Population and samples

Data used for these analyses were obtained from the adult subsample (subjects 15 years and older) of the first 4 waves of the South African National Income Dynamics Study (NIDS).[16]

The NIDS is an ongoing nationally representative panel survey of 28,255 South Africa’s residents. The baseline data collection was conducted in 2008, when a two-stage cluster sample design was used to randomly select about 7,300 households across 400 primary sampling units, stratified by district council (a second level administrative division of South Africa’s territory into 52 areas). All available adult subjects belonging to the selected households were eligible to be interviewed and administered the adult questionnaire. In the following three waves of data collection so far (in 2010–2011, 2012 and 2014–2015) the same individuals (continuing sample members) were recontacted and administered the same questionnaire. In addition, all adults belonging to the same household of the continuing sample members at the moment of the interview (temporary sample members) were also interviewed. Their data were made available in the dataset for cross-sectional rather than longitudinal analyses. A total of 17,372 adults were interviewed at baseline, with responses rate of 67.2% at the household level and 93.3% at the individual level. In the following three waves the total number of individual interviewed (including both continuing and temporary sample members) was respectively 18,732 (cross-sectional response 78.5%), 21,414 (73.9%) and 24,353 (66.7%).

Further details of the sampling strategy and realization are provided in the methodological article by Woolard et al. and in the survey User Manual.[17,18] These include methods of calculation and calibration of the sampling weights provided with the datasets to take into account the sampling design, unequal response rates across population strata and the presence of temporary sample members.

The NIDS study, whose anonymised datasets are publicly available for research purposes, has been granted ethics approval by the Commerce Faculty Ethics Committee at the University of Cape Town. The analysis presented here was further approved by the Human Research Ethics Committee of the Faculty of Health Sciences, University of Cape Town (HREC REF:506/2013). This study used data from NIDS wave 1 dataset v.6.1, wave 2 v.3.1, wave 3 v.2.1and wave 4 v.1.2.[19–22]

### Measures

#### Sociodemographic variables

Age in years was categorised into 6 groups. Racial ascription was self-defined by participants along the lines of the historical “population group” classification used in South Africa during apartheid: Asian (mainly Indian descent), Black (or African), Coloured (wide grouping of people of mixed ancestry) and White (mainly European descent). Race in this sense is closely and enduringly correlated with socioeconomic status in South Africa but continues to have independent predictive value as a measure of health inequity. The term “racial ascription” captures the historical and social nature of this classification. Education, as one indicator of socioeconomic status, was defined as Primary, Secondary, Tertiary and None according to years of completed schooling. Place of residence was categorised as urban/rural according to Statistics South Africa's Census 2001.[23]

#### Blood pressure

Individual values of systolic and diastolic BP constitute the main outcome measures of the study. Duplicate measurements of BP were taken in the left arm, after the participant was seated for at least 5 minutes, by using automated oscillometric devices (Omron M7 BP Monitor, factory calibrated). The devices were validated according to international protocols,[24] and used with their standard multi-size cuffs. By applying the cut-offs used by the Global Burden of Metabolic Risk Factors of Chronic Diseases Collaborating Group, SBP readings were retained in the datasets if ≥70 mm Hg and <270 mm Hg, and DBP readings were retained if >30 mm Hg and <180 mm Hg.[25] Differences between SBP and DBP < 15 mm Hg were also considered implausible, and, in agreement with common practice in epidemiological studies on blood pressure (including the three editions of the WHO Demographic and Health Survey carried out in South Africa [23]) these pairs were excluded from the available measurements.

#### Other measurements

Duplicate measures of weight and height were recorded, with a third measure taken if their difference was greater than 0.5 kg or 0.5 cm respectively. Excluding measures with implausible values (height <60 cm or >230 cm, weight <30 kg or >250 kg), the average of the available readings was considered as the subject’s true value and used to calculate body mass index (BMI) in kg/m^2^. For descriptive purposes, BMI was then categorised in four classes according to the World Health Organization's cut-off points.[26] Waist circumference was measured twice and, after exclusion of implausible values (waist circumference <30 cm or >200 cm), the average of the available readings taken as the subject’s true value.

Current smoking, any alcohol consumption, use of antihypertensive medication, past diagnosis of hypertension by a health professional and history of cardiovascular disease (i.e. any episode of stroke or heart attack) were self-reported by subjects in response to direct questions.

### Statistical analyses

All analyses were conducted separately by gender, based on prior evidence that most of the relationships between the variables involved in our analyses differ substantially by gender.

Observed values of SBP and DBP were statistically adjusted for seasonality that could have biased between-wave comparisons and trend estimates, given the large differences in the distribution of data collection during the year and the magnitude of seasonal effects observed in the South African population.[27] Season-adjusted values of each reading ─ centred at the average period of data collection at baseline ─ were predicted with a linear model including as covariates (1) a cosinor function, (2) a linear spline with a single knot at age = 55 years to represents age, and (3) their interaction. Cosinor functions are frequently used in epidemiological studies to model seasonal patterns, and they have been previously applied to the study of seasonal variations of blood pressure.[28] The spline formulation of the age covariate reflects substantial epidemiological evidence that both SBP and DBP rise during childhood and adulthood until the 6^th^ decade of life, after which SBP continues usually to rise at a slower pace, while DBP tends to remain constant or to decline.[29] The interaction terms were included on account of evidence that seasonal effects vary by age.[27] The averages of the season-adjusted duplicate readings of SBP and DBP were considered as the true values of the individual BP.

To adjust the trend estimates for the effect of antihypertensive treatment and recover the values that would have been observed in the hypothesis of no treatment (counterfactual values), we adapted the censored regression approach described by Konigorski et al.[30] Ordinary methods of adjustment (i.e. introducing treatment status as a covariate in regression models) are well known to produce biased estimates. This is because subjects are initiated on treatment depending on their pre-treatment values of BP and consequently treatment status cannot be considered independent of the underlying untreated BP.[31] The non-independence of treatment status from the outcome violates the basic assumption underlying the common estimators of generalised regression models, producing biased estimates of the regression coefficients. A detailed description of the estimation procedure used to recover counterfactual values can be found in the supplementary material as S1 File.

The averages of the predicted duplicated readings of SBP and DBP were considered as the true values of the individual BP in the absence of treatment.

Observed and counterfactual season-adjusted individual values were used to calculate cross-sectional estimates of mean SBP and BP and prevalence of uncontrolled hypertension and their trends. The role of treatment was analysed by comparing observed and counterfactual values.

For trend estimation, individual measurements in each wave were considered concentrated at the median month of data collection (April 2008, September 2010, August 2012 and January 2015 for the successive waves). The number of subjects with uncontrolled hypertension was calculated by multiplying the estimated prevalence in each period by the population totals calculated by linear interpolation from the mid-year population estimates published by Statistics South Africa, assumed error-free.[32]

To assess the contribution of the changing distribution of bio-behavioural risk factors in explaining the observed trends, we used linear/logistic regression to further adjust SBP and DBP and trends of uncontrolled hypertension for BMI, waist circumference, alcohol use and smoking. The fully adjusted trends were compared with those adjusted for season alone.

The complex sampling design of the NIDS (which includes clustering, stratification and unequal probability of selection) was taken into account in the analyses. Sampling weights were integrated into all analyses through the use of weighted maximum likelihood estimators. Standard errors were calculated by bootstrapping the whole procedure above (including the prediction of season-adjusted observed values and the counterfactual, untreated BP values) and applying the usual methods to combine the replicated estimates. Four hundred sets of replicated bootstrap weights were generated, taking into account the clustered and stratified structure of the sample as described by Lumley.[33]

Multiple imputation with chained equations was used to deal with the presence of non-negligible proportions of missing data.[34] We generated twenty imputed datasets and assessed the convergence of the imputation algorithm by plotting mean and variance of each imputed variable against the iteration number and visually inspecting the correct stabilization and random mixing of the streams. We repeated the analyses described above in each dataset and combined the results with Rubin’s rules.[35] The combination of bootstrap with multiple imputation has been studied by Schomaker and Heumann, and the results of their simulations show that the procedure is able to provide valid inference in realistic settings.[36]

### Sensitivity analysis

The model used to estimate counterfactual values of SBP and DBP involved a series of assumptions not directly testable with the data. To explore the robustness of our findings with respect to this extra level of uncertainty we repeated the estimation by introducing large random perturbations (uniformly distributed in the interval +/- 5 mm Hg) in the predicted individual values and compare the results with the original ones.

All analyses were conducted with R statistical software v. 3.2.[37] The additional packages Mice, Survey v. 3.3 and Survival v. 2.38 were used to generate the multiple imputed datasets, to calculate the replicated bootstrap weights and to estimate the censored regression models, respectively.[38–40]

## Results

Demographic and bio-behavioural characteristics of the four samples analysed in this study are described in Table 1.

**Table 1:** Unweighted sample descriptive statistics.

	NIDS,2008			NIDS,2010–11			NIDS,2012			NIDS,2014–15		
Variable	n	Med/ Percent	IQR/ Frequency	n	Med/ Percent	IQR/ Frequency	n	Med/ Percent	IQR/ Frequency	n	Med/ Percent	IQR/ Frequency
**Men**	18,617	43.8%	8,143	19,307	43.1%	8,322	21,810	43.0%	12,389	24,856	43.15%	10,725
**Age class**	18,541			19,293			21,780			24,808		
**15–24**		30.7%	5,690		33.1%	6,387		32.4%	7,052		31.6%	7,839
**25–34**		19.7%	3,657		20.1%	3,884		21.2%	4,620		22.7%	5,642
**35–44**		16.6%	3,082		15.4%	2,967		15.2%	3,309		14.8%	3,678
**45–54**		13.8%	2,567		13.1%	2,525		12.9%	2815		12.5%	3,111
**55–64**		9.5%	1,766		9.4%	1,815		9.5%	2,067		9.6%	2,367
**65+**		9.6%	1,779		8.9%	1,715		8.8%	1,917		8.7%	2,162
**Racial ascription**	18,617			19,306			21,810			24,856		
**Black**		76.6%	14,254		81.8%	15,793		81.2%	17,718		82.5%	20,500
**Coloured**		15.4%	2,859		13.6%	2,632		14.4%	3,133		14.1%	3,509
**White**		6.3%	1,182		3.3%	638		3.2%	694		2.4%	606
**Asian**		1.7%	322		1.3%	243		1.2%	265		1.0%	241
**Education**	18,510			19,270			21,725			24,763		
**None**		13.1%	2,417		12.2%	2,357		10.8%	2,358		8.8%	2,185
Primary*		23.9%	4,434		22.4%	4,316		20.9%	4,529		19.0%	4,706
Secondary*		53.8%	9,950		55.8%	10,744		56.7%	12,312		56.9%	14,094
Tertiary*		9.2%	1,709		9.6%	1,853		11.6%	2,526		15.35	3,778
**Urban**	18,617	50.5%	9,395	19,228	46.3%	8,897	21,810	47.4%	10,342	24,856	49.4%	12,287
**Current smoking**	15,507	21.1%	3,277	16,775	15.8%	2,645	19,901	14.6%	2,898	22,738	18.8%	4,226
**Current alcohol use**	15,504	24.3%	3,767	16,735	20.5%	3,437	18,664	23.5%	4,386	22,737	28.7%	6,532
**Waist circ. [cm]**	13,970	83.1	[74.2;95.4]	15,146	82.0	[72.0;96.0]	18,262	86.0	[76.0;98.7]	22,402	84.8	[74.8;98.9]
**BMI [kg/m2]**	13,885	24.4	[20.9;29.7]	15,122	25.0	[21.4;30.3]	18,317	25.0	[21.5;29.9]	22,324	24.8	[21.0;30.6]
BMI category**	13,885			15,122			18,317			22,324		
**Underweight**		6.8%	947		5.7%	863		3.9%	722		5.3%	1,193
**Normal weight**		47.2%	6,550		44.4%	6,714		46.0%	8,428		45.6%	10,171
**Overweight**		22.1%	3,066		23.9%	3,614		25.3%	4,632		22.1%	4,926
**Obese**		23.9%	3,322		26.0%	3,931		24.8%	4,535		27.0%	6,034
**CVD history**	18,617	3.7%	689	19,307	2.5%	481	21,810	3.5%	757	24,856	2.6%	649
**Diagnosis of hypertension**	17,141	15.7%	2,686	18,480	12.0%	2,216	21,282	16.4%	3,496	21,915	10.6%	2,330
**Antihypertensive treatment**	16,846	11.5%	1,932	18,268	9.2%	1,690	21,005	12.0%	2,527	23,659	13.9%	3,288
**First systolic reading [mm Hg]**	13,792	123	[111;139]	14,745	122	[110;136]	18,318	121	[110;135]	22,455	121	[109;135]
**Second systolic reading [mm Hg]**	13,732	121	[109;136]	14,622	120	[109;134]	18,306	119	[108;133]	22,446	119	[107;133]
**First diastolic reading [mm Hg]**	13,810	80	[71;90]	14,730	79	[71;89]	18,320	80	[72;90]	22,466	79	[71;89]
**Second diastolic reading [mm Hg]**	13,713	79	[70;89]	14,644	78	[70;88]	18,308	79	[71;89]	22,455	78	[70;88]

n = number of not missing values; Med = median; IQR = interquartile range. BMI = body mass index; CVD cardiovascular disease.

* Some or completed. Tertiary education includes any further training after completion of secondary education.

** Underweight: BMI < 18.5 Kg/m^2^; Normal Weight: 18.5 Kg/m^2^ ≤ BMI < 25 Kg/m^2^; Overweight: 25 Kg/m^2^ ≤ BMI < 30 Kg/m^2^; Obese: BMI > 30 Kg/m^2^.

Note that the total number of non-missing values for some demographic variables in the NIDS samples exceeds the number of subjects actually interviewed reported in the description of survey response rates in the article. This is because the NIDS datasets incorporate demographic information on subjects not interviewed recovered from the household roster. The sampling weights provided with the datasets are calculated taking into account these individuals.

With reference to the South African general population in the same period, in all samples, women, older age groups and rural dwellers were moderately overrepresented. Whites were more severely under-represented, owing to their low response rate at baseline and greater loss to follow-up in the subsequent waves. Their sample proportions lay between 6.3% in 2008 and 2.4% in 2015, as opposed to. 9.4% to 8.2% respectively according to census data. Further, compared to the estimates from the general household survey carried out annually by Statistics South Africa,[32] the samples included higher proportions of subjects with low education levels (primary or no formal education). Prevalences of self-reported previous diagnosis of hypertension and antihypertensive treatment were also higher than the corresponding estimates from the general household survey.[32]

### Observed trends in blood pressure, prevalence of uncontrolled hypertension and self-reported antihypertensive medication use

Season-adjusted estimates of mean SBP and DBP and prevalence of uncontrolled hypertension in each wave are shown in Table 2.

**Table 2:** Season-adjusted mean blood pressure and prevalence of uncontrolled hypertension in the South African adult population (aged 15 years and over) by age group and gender, 2008–2015. Estimates and standard errors.

	Women			Men		
Age group [years] and period of data collection	SBP	DBP	HTN	SBP	DBP	HTN
	[mm Hg]	[mm Hg]	[%]	[mm Hg]	[mm Hg]	[%]
**15–24**						
** 2008**	110.7 (0.53)	73.1 (0.44)	6.7 (0.92)	117.5 (0.52)	73.3 (0.40)	9.9 (1.18)
** 2010–11**	108.3 (0.58)	71.9 (0.47)	6.2 (0.87)	115.3 (0.61)	72.9 (0.43)	8.2 (0.98)
** 2012**	107.4 (0.54)	72.8 (0.42)	5.9 (0.74)	114.8 (0.63)	73.3 (0.44)	9.0 (1.06)
** 2014–15**	104.6 (0.44)	71.6 (0.33)	3.5 (0.62)	114.6 (0.47)	72.9 (0.35)	6.8 (0.88)
**25–34**						
** 2008**	116 (0.58)	77.9 (0.4)	14.1 (1.33)	122.1 (0.78)	77.5 (0.54)	17.5 (1.91)
** 2010–11**	113.8 (0.8)	76.8 (0.58)	12.7 (1.52)	120.9 (0.95)	77.4 (0.62)	16.2 (1.68)
** 2012**	112.8 (0.6)	77.6 (0.5)	14.3 (1.39)	119.9 (0.69)	78.7 (0.52)	15.9 (1.79)
** 2014–15**	110.1 (0.48)	76.8 (0.38)	11.3 (1.08)	120.7 (0.67)	78.6 (0.45)	18.9 (1.95)
**35–44**						
** 2008**	124.4 (0.86)	83.3 (0.61)	30.2 (2.08)	125.8 (0.84)	80.6 (0.60)	25.8 (2.14)
** 2010–11**	119.9 (0.88)	80.5 (0.64)	22.7 (1.90)	124.5 (1.07)	80.7 (0.74)	25.4 (2.77)
** 2012**	119.4 (0.77)	81.7 (0.53)	21.6 (1.63)	125.5 (1.14)	82.1 (0.75)	25.0 (2.74)
** 2014–15**	117.4 (0.68)	81.2 (0.49)	21.8 (1.70)	125.0 (0.88)	82.3 (0.60)	25.3 (2.19)
**45–54**						
** 2008**	131.2 (0.99)	85.9 (0.60)	39.8 (2.26)	133.5 (1.05)	84.4 (0.66)	36.7 (2.61)
** 2010–11**	128.7 (1.15)	84.9 (0.70)	36.9 (2.72)	130.6 (1.25)	83.9 (0.89)	34.5 (3.15)
** 2012**	127.2 (1.03)	85.0 (0.62)	35.1 (2.43)	129.8 (1.06)	84.5 (0.70)	35.4 (2.79)
** 2014–15**	126.5 (0.92)	85.1 (0.62)	33.8 (2.03)	127.7 (1.06)	84.1 (0.63)	33.1 (2.71)
**55–64**						
** 2008**	140.8 (1.30)	88.8 (0.78)	53.2 (2.54)	140.7 (1.53)	87.1 (0.89)	49.9 (3.14)
** 2010–11**	137.2 (1.56)	86.5 (0.96)	44.9 (2.90)	138.2 (1.91)	85.4 (1.13)	44.4 (3.95)
** 2012**	136.3 (1.51)	86.7 (0.84)	46.1 (2.84)	135.8 (1.61)	86.1 (0.99)	43.2 (3.89)
** 2014–15**	133.0 (1.30)	86.0 (0.78)	39.8 (2.52)	136.7 (1.55)	85.7 (0.78)	43.5 (3.49)
**65+**						
** 2008**	148.0 (1.48)	90.2 (0.78)	61.8 (2.75)	146.0 (1.91)	87.5 (1.04)	58.0 (3.62)
** 2010–11**	144.8 (1.94)	89.0 (1.10)	57.4 (3.92)	145.1 (2.67)	87.2 (1.37)	58.4 (4.54)
** 2012**	144.4 (1.88)	87.4 (0.92)	56.1 (3.15)	145.1 (2.06)	87.8 (1.08)	57.8 (4.12)
** 2014–15**	140.2 (1.39)	84.7 (0.89)	49.3 (2.86)	141.3 (1.60)	84.7 (0.93)	47.4 (3.40)
**15+**						
** 2008**	122.8 (0.44)	80.5 (0.31)	25.6 (0.83)	125.7 (0.48)	79.0 (0.33)	24.0 (0.98)
** 2010–11**	120.0 (0.50)	79.0 (0.36)	22.5 (0.88)	123.8 (0.71)	78.6 (9.46)	22.4 (1.24)
** 2012**	119.4 (0.49)	79.7 (0.33)	22.7 (0.78)	123.5 (0.61)	79.6 (0.39)	22.7 (1.19)
** 2014–15**	117.0 (0.44)	79.0 (0.25)	20.2 (0.78)	123.3 (0.44)	79.4 (0.30)	22.3 (1.01)

SBP/DBP = Average systolic/diastolic blood pressure; HTN = prevalence of uncontrolled hypertension. Standard errors in brackets.

Among women, with a few exceptions, mean SBP and DBP and prevalence of uncontrolled hypertension decreased consistently in successive waves in all age groups. In the female population as a whole, linear regression slopes for mean SBP and DBP were both negative and statistically significant. The magnitude was -0.81 mm Hg/year (95% CI: -0.95 to -0.67) for SBP and -0.19 mm Hg/year (95% CI -0.29 to -0.09) for DBP. The odds of uncontrolled hypertension estimated by the logistic regression model also showed a significant downward trend: each year the odds were reduced by 4.0% compared to the previous (95% CI: -5.5% to -2.5%)

Among men a consistent decrease was present for SBP, with overall linear regression slope -0.34 mm Hg/year (95% CI -0.50 to -0.18), while DBP showed a modest and not statistically significant upward trend (0.10 mm Hg/year; 95% CI: -0.02 to 0.20). The odds of hypertension tended to decrease slightly over time, by 1.0%/year (95% CI: -3.2% to 0.0%). In each survey, the percentage of subjects taking antihypertensive medication (Table 3) increased with age, and within each age group it was higher among women than among men. In both genders, the total proportion on medication calculated across all age groups was similar between the first two survey waves but increased across subsequent waves particularly in the age strata > 34 years. Between the first and last survey waves, the estimated proportion of treated subjects increased by 34.3% among women and by 54.7% among men.

**Table 3:** Proportion of subjects on antihypertensive treatment in the South African adult population by age group and gender, 2008–2015. Estimates and standard errors.

	Women				Men			
Age group [years]	2008	2010–11	2012	2014–15	2008	2010–11	2012	2014–15
**15–24**	0.8 (0.25)	0.3 (0.11)	0.5 (0.17)	0.4 (0.13)	0.2 (0.10)	0.2 (0.08)	0.1 (0.10)	0.4 (0.22)
**25–34**	3.0 (0.73)	2.8 (1.03)	2.3 (0.49)	3.4 (0.80)	0.6 (0.24)	0.7 (0.42)	0.5 (0.22)	0.9 (0.32)
**35–44**	13.2 (1.58)	8.9 (1.22)	12.0 (1.28)	14.6 (1.59)	3.5 (0.89)	2.9 (0.88)	4.7 (1.34)	6.1 (1.43)
**45–54**	25.8 (1.74)	21.1 (1.74)	26.4 (1.75)	30.6 (1.96)	10.3 (1.55)	8.7 (1.57)	11.7 (1.58)	14.6 (1.92)
**55–64**	35.4 (2.46)	34.7 (2.74)	43.2 (2.43)	48.3 (2.20)	21.4 (2.82)	18.2 (2.56)	28.3 (3.18)	27.9 (2.64)
**65+**	39.8 (2.61)	40.7 (3.65)	50.9 (3.11)	59.6 (2.70)	24.9 (2.95)	29.5 (3.66)	33.4 (3.49)	40.6 (3.42)
**15+**	13.1 (0.65)	11.7 (0.71)	14.8 (0.58)	17.6 (0.66)	5.3 (0.42)	5.0 (0.46)	6.9 (0.50)	8.2 (0.59)

Standard errors in brackets.

### Counterfactual scenario in the absence of antihypertensive treatment

**Fig 1:**
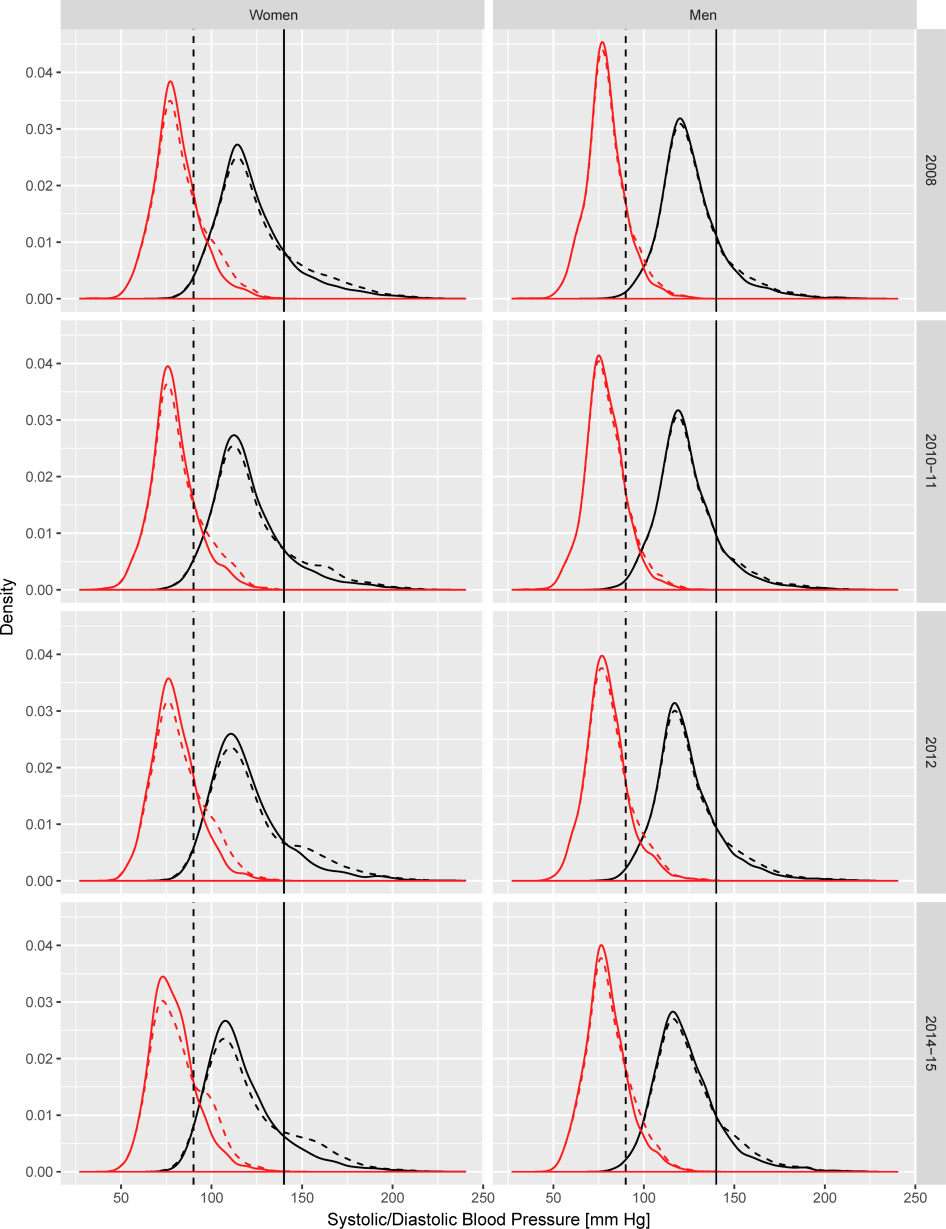
Season-adjusted observed and counterfactual distribution of systolic and diastolic blood pressure in the South African adult population (15 years and over), by gender. Solid lines = observed distribution; Dashed lines = distribution in absence of treatment. The curves on the left side refer to DBP, those on the right side refer to SBP. Vertical lines represent the cut-off for diagnosis of diastolic (dashed line) and systolic (solid line) hypertension.

Fig 1 compares the observed distribution of SBP and DBP in each period with the modelled counterfactual distribution that would have been observed in the absence of antihypertensive treatment.

As expected considering the current South African guidelines for the prescription of antihypertensive drugs, the estimated effect of treatment was a change affecting only the right tail of the distributions, roughly above the diagnostic thresholds of 90/140 mm Hg. This proportion of hypertensive individuals estimated to occur in the absence of treatment tended to increase over time, especially among women.

**Fig 2:**
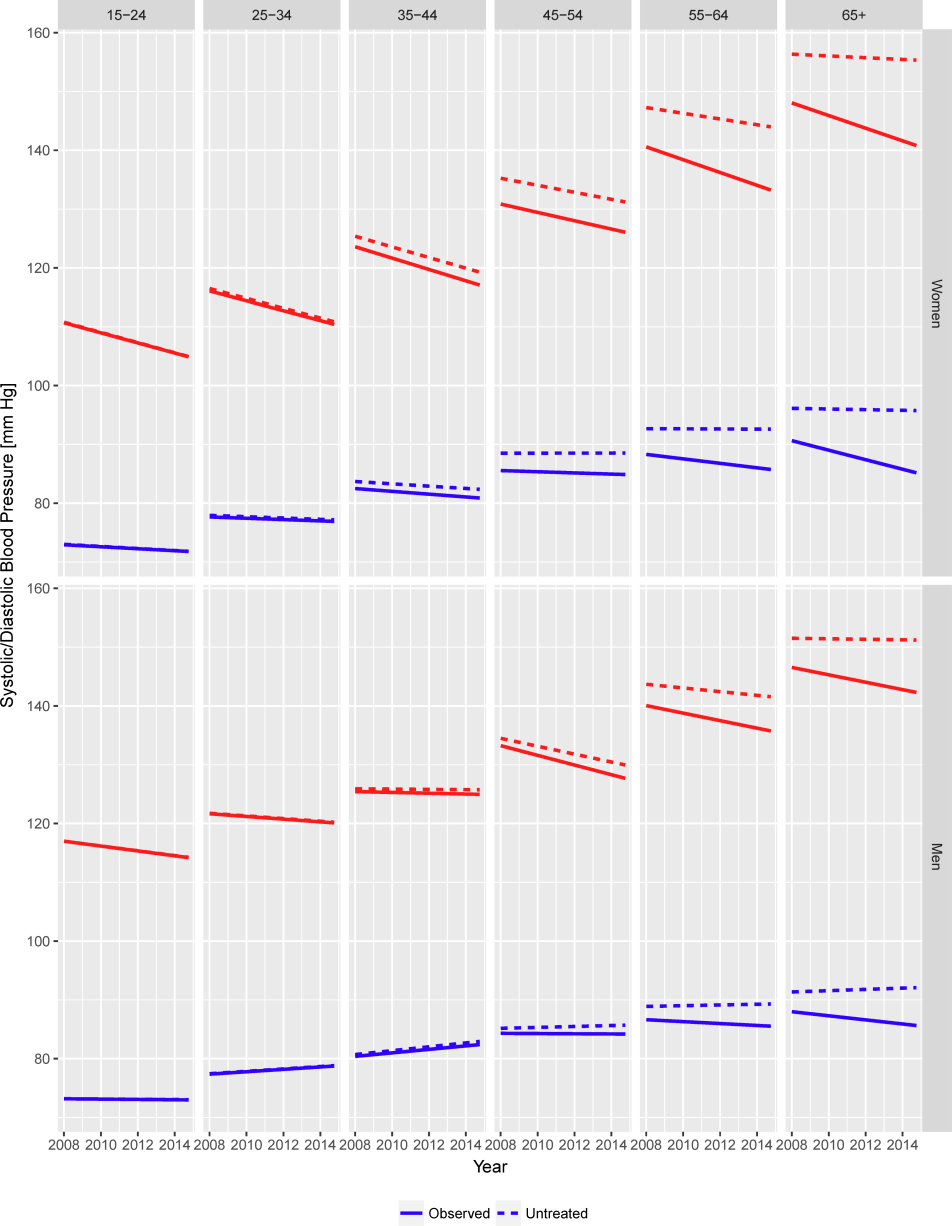
Season-adjusted observed and counterfactual linear trends in mean systolic and diastolic blood pressure in the South African adult population (aged 15 years and over), by age group and gender.

The effect of treatment on the linear trends of SBP and DBP is shown in Fig 2, separately for each age category.

In the 15–24 years age category (where the proportion of subjects on treatment was extremely small at each time point) observed and counterfactual trends in the absence of treatment coincided. Among subjects aged 25 to 44 years the population effects of treatment became detectable but with a magnitude of little, if any, practical relevance.

Moving towards older age categories, however, the contribution of treatment to the observed trends became increasingly apparent. In both genders, trends in untreated DBP were estimated as flat or positive among subjects 45 years and older, suggesting treatment effects as a major driver of the decreases actually observed. Counterfactual SBP trends were similarly attenuated compared to the observed ones, and among subjects 65 years and older any significant favourable trend in SBP disappeared once adjusted for the estimated treatment effect.

In the female population, the counterfactual trend (absence of treatment) for SBP was still downward but with a less steep regression slope, i.e. -0.62 mm Hg/year (95% CI: -0.77 to -0.48), compared to the unadjusted slope of -0.81 mm Hg/year. The adjusted regression slope for DPB was also flattened, relative to the unadjusted slope of -0.19 mm Hg/year, to -0.06 mm Hg/year (95% CI: -0.15 to 0.04), no longer statistically significant.

Similarly, among men, the counterfactual linear regression slope for SBP was closer to the null at -0.24 mm Hg/year (95% CI: -0.38 to -0.09) relative to the unadjusted value of -0.34 mm Hg/year. In the case of the observed upward trend in DPB, the counterfactual slope was steeper at 0.17 mm Hg/year (95% CI: 0.06 to 0.27) than the unadjusted value of 0.10 mm Hg/year.

Numerical values of the age-specific observed and counterfactual trends are reported in Table 4.

**Table 4:** Trends in mean blood pressure and prevalence of uncontrolled hypertension in the South African adult population (aged 15 years and over) between 2008 and 2014–15. Comparison between observed and counterfactual trends in absence of antihypertensive treatment, by age group and gender. (Regression coefficients and standard errors).

	Women						Men					
	Systolic [mm Hg / year]		Diastolic [mm Hg / year]		HTN [% / year]		Systolic [mm Hg / year]		Diastolic [mm Hg / year]		HTN [% / year]	
Age group [years]	βo (se)	βu (se)	βo (se)	βu (se)	ORo (se)	ORu (se)	βo (se)	βu (se)	βo (se)	βu (se)	ORo (se)	ORu (se)
**15–24**	-0.86 (0.08)	-0.86 (0.08)	-0.17 (0.07)	-0.18 (0.07)	0.92 (0.02)	0.92 (0.02)	-0.41 (0.1)	-0.41 (0.1)	-0.03 (0.08)	-0.02 (0.08)	0.95 (0.03)	0.95 (0.03)
**25–34**	-0.84 (0.10)	-0.83 (0.10)	-0.11 (0.07)	-0.11 (0.08)	0.97 (0.02)	0.98 (0.02)	-0.23 (0.12)	-0.23 (0.12)	0.20 (0.08)	0.20 (0.08)	1.01 (0.03)	1.01 (0.03)
**35–44**	-0.96 (0.16)	-0.90 (0.17)	-0.24 (0.12)	-0.20 (0.12)	0.94 (0.02)	0.96 (0.02)	-0.07 (0.18)	-0.02 (0.17)	0.30 (0.11)	0.33 (0.11)	1.00 (0.02)	1.00 (0.02)
**45–54**	-0.71 (0.17)	-0.6 (0.18)	-0.1 (0.11)	0.00 (0.12)	0.96 (0.02)	0.98 (0.02)	-0.82 (0.2)	-0.67 (0.21)	-0.02 (0.12)	0.08 (0.13)	0.98 (0.02)	1.00 (0.02)
**55–64**	-1.09 (0.24)	-0.49 (0.23)	-0.38 (0.14)	-0.01 (0.14)	0.93 (0.02)	1.00 (0.02)	-0.64 (0.29)	-0.31 (0.32)	-0.16 (0.17)	0.06 (0.19)	0.96 (0.03)	0.99 (0.03)
**65+**	-1.07 (0.29)	-0.15 (0.27)	-0.81 (0.17)	-0.06 (0.15)	0.93 (0.02)	1.04 (0.03)	-0.63 (0.36)	-0.04 (0.35)	-0.35 (0.20)	0.11 (0.20)	0.94 (0.03)	1.00 (0.03)
**15+**	-0.81 (0.07)	-0.62 (0.07)	-0.19 (0.05)	-0.06 (0.05)	0.96 (0.01)	0.99 (0.01)	-0.34 (0.08)	-0.24 (0.08)	0.10 (0.05)	0.17 (0.05)	0.99 (0.01)	1.00 (0.01)

Βo/βu = linear regression slope for the observed/counterfactual trend; ORo/ORu = relative odds of uncontrolled hypertension for each successive year of observation in the observed/counterfactual scenario.

**Fig 3:**
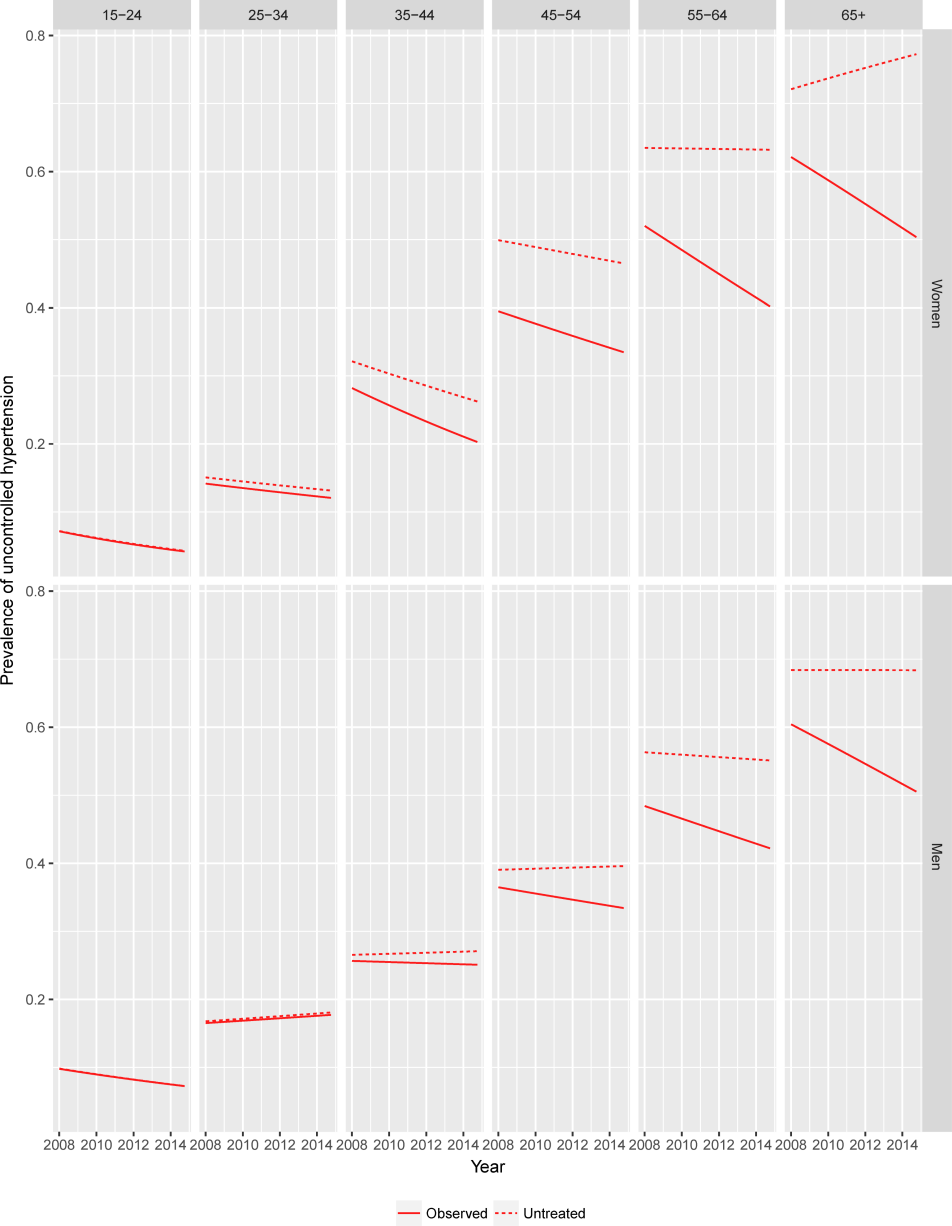
Season-adjusted observed and counterfactual trends in the prevalence of uncontrolled hypertension in the South African adult population (15 years and over) by age group and gender. The lines in the figure represent predicted prevalence of uncontrolled hypertension estimated by logistic models with observed and counterfactual individual values as outcomes.

Table 4 also compares observed and counterfactual trends in the prevalence of uncontrolled hypertension. The same information is graphically depicted in Fig 3.

The effect of treatment was evident in both genders in the older age classes, with counterfactual trends of opposite sign to those observed among women 55 years old and over and among men 45 years old and over. Specifically, among women the observed decreasing trend in the odds of uncontrolled hypertension was almost completely offset in the counterfactual scenario (from 4.0%/year to 0.9%/year). Among men the counterfactual trend in the odds of hypertension was statistically flat (odds ratio 1.0).

Finally, Table 5 shows the differences in the observed and counterfactual prevalence of hypertension and number of subjects affected for each data point.

**Table 5:** Observed and counterfactual prevalence of uncontrolled hypertension and number of subjects affected in the South African population (aged 15 years and over) by gender, between 2008 and 2014–15. Estimates and standard errors.

	Women				Men			
	2008	2010–11	2012	2014–15	2008	2010–11	2012	2014–15
	**Prevalence of uncontrolled hypertension**							
**Observed [%]**	25.6 (0.83)	22.5 (0.88)	22.7 (0.78)	20.2 (0.78)	24.0 (0.98)	22.4 (1.24)	22.7 (1.19)	22.3 (1.01)
**Untreated [%]**	30.4 (0.89)	27.0 (0.96)	28.9 (0.84)	28.4 (0.81)	26.0 (1.01)	24.1 (1.26)	25.8 (1.21)	25.8 (1.07)
**Difference [%]**	4.8 (0.41)	4.5 (0.44)	6.2 (0.51)	8.2 (0.29)	2.0 (0.29)	1.7 (0.29)	3.0 (0.40)	3.5 (0.41)
	**Number of subjects affected**							
**Observed [n]**	3951085	3740999	3933098	3726101	4189808	4047429	4258233	4429340
**Untreated [n]**	4691913	4539079	5007337	5238676	4538959	4354600	4839754	5124528
**Difference [n]**	740828	798080	1074239	1512575	349151	307171	581521	695188

Difference represents the excess prevalence/number of uncontrolled hypertensive subjects that would have been observed in absence of treatment. Standard errors in brackets.

According to our simulation, in absence of treatment, in the South African population as a whole we would have observed 1,089,979 (95% CI 856,368 to 1,323,590) more uncontrolled hypertensive subjects in 2008; 1,105,251 (95% CI: 845,054 to 1,365,448) in 2010–11; 1,655,760 (95% CI: 1,326,284 to 1,985,234) in 2012; and 2,207,763 (95% CI: 1,947,288 to 2,468,238) in 2014–15.

### Adjustment for bio-behavioural risk factors

During the study period, the mean age of the South African population increased by an average 0.13 years for each year among women (95% CI: 0.05 to 0.21), and 0.10 years for each year among men (95% CI: 0.03 to 0.18).

Age- and gender-specific estimated linear trends in BMI, waist circumference, prevalence of alcohol users and smokers during the study period are reported in Table 6.

**Table 6:** Age- and gender-specific linear trends in BMI, waist circumference, prevalence of alcohol users and smokers in the South African adult population (aged 15 years and over), between 2008 and 2014–15. Estimates and 95% confidence intervals.

Age category [years]	BMI [kg/m^2^ per year]	Waist circ. [cm per year]	Prevalence of alcohol users [% per year]	Prevalence of smokers [% per year]
**Females**				
**15–24**	0.00 (-0.05;0.06)	0.47 (0.26;0.68)	0.59 (0.12;1.07)	-0.01 (-0.31;0.29)
**24–34**	0.15 (0.06;0.23)	0.94 (0.69;1.20)	1.87 (1.24;2.49)	-0.35 (-0.77;0.07)
**35–44**	0.11 (-0.01;0.23)	0.95 (0.64;1.26)	0.66 (-0.15;1.47)	-0.39 (-1.00;0.23)
**45–54**	0.12 (0.00;0.25)	0.92 (0.55;1.29)	0.44 (-0.2;1.07)	0.39 (-0.43;1.21)
**55–64**	0.17 (0.02;0.33)	1.11 (0.65;1.58)	-0.34 (-1.17;0.48)	-0.11 (-0.70;0.49)
**65+**	-0.05 (-0.17;0.07)	0.93 (0.56;1.31)	-0.23 (-0.99;0.53)	-0.60 (-1.16;-0.05)
**15+**	0.10 (0.06;0.14)	0.88 (0.7;1.06)	0.73 (0.39;1.08)	-0.16 (-0.38;0.06)
**Males**				
**15–24**	-0.07 (-0.12;-0.01)	0.35 (0.17;0.52)	1.06 (0.49;1.63)	-0.10 (-0.66;0.47)
**24–34**	-0.02 (-0.11;0.06)	0.64 (0.24;1.05)	2.02 (1.15;2.88)	0.20 (-0.6;0.99)
**35–44**	-0.05 (-0.16;0.06)	0.47 (0.16;0.79)	0.22 (-0.72;1.16)	-0.96 (-1.84;-0.08)
**45–54**	-0.03 (-0.15;0.09)	0.72 (0.36;1.08)	1.24 (0.16;2.31)	-0.42 (-1.54;0.69)
**55–64**	0.00 (-0.19;0.20)	1.14 (0.57;1.70)	-0.46 (-1.68;0.76)	0.14 (-1.08;1.36)
**65+**	0.12 (-0.06;0.30)	1.22 (0.71;1.73)	-0.59 (-2.16;0.98)	-0.22 (-1.46;1.02)
**15+**	-0.01 (-0.06;0.03)	0.67 (0.48;0.86)	1.06 (0.67;1.45)	-0.11 (-0.45;0.24)

Confidence intervals in brackets.

In both genders, waist circumferences and the prevalence of alcohol users increased during the study period. The increase of waist circumference was consistent across all age categories, while the prevalence of alcohol users increased significantly only in the youngest age categories (15 to 34 years). In both genders the overall prevalence of smokers did not change significantly with the exception of older women, where the estimate showed a significant decrease. Among women, BMI increased across almost all age categories with the exception of the last (65 years and over), albeit at a slower pace compared to waist circumference. Conversely, BMI did not change significantly among men of any age category. A rapid increase in waist circumference accompanied by slower or null increases in BMI is a phenomenon increasingly observed in various populations, both in adults and children.[41–43]

Regarding the impact on BP levels, the trends described above are substantially unfavourable. This is especially the case of waist circumference across all age categories, alcohol use in subjects younger than 55 years and, limited to women, BMI. For the inverse relationship between smoking and body weight usually observed in cross-sectional studies,[44] the downward trend in smoking, albeit not significant, can also be considered as unfavourable.

Statistical adjustment for bio-behavioural risk factors, i.e. removing the effect of any trend in these factors, thus resulted in steeper decreases in the average values of SBP and DBP than those actually observed. In other words, if it weren’t for the unfavourable trends in these risk factors, the decline in blood pressure would have been even greater than observed. Among women, the regression slope for SBP steepened from -0.81 mm Hg/year to -0.96 mm Hg/year (95% CI: -1.04 to -0.87) and that for DBP from—0.19 mm Hg/ year to -0.29 mm Hg/year (95% CI -0.35 to -0.24). Similarly, the decreasing trend in the odds of uncontrolled hypertension was accentuated after adjustment, at 6.4%/year (95%CI: 4.6% to 8.1%). Among men, adjustment for biobehavioural risk factors produced a similar pattern of change to that in women. The regression slope for SBP steepened from -0.34 mm Hg/year to -0.47 mm Hg/year (95% CI: -0.57 to -0.37) while that for DBP fell slightly from 0.10 to 0.00 mm Hg/year (95% CI -0.06 to 0.06), but remained null for trend. The adjusted overall odds ratio decreased by 2.8%/year (95%CI: 0.69% to 4.9%).

### Sensitivity analysis

The introduction of the random perturbations described in the methods section in the predicted counterfactual values of SBP and DBP (amounting to more than 25% of the average treatment effect in the treated) did not modify the overall conclusions regarding the effect of treatment in explaining part of the observed decrease in BP and prevalence of hypertension across age categories.

Counterfactual trends in the odds of uncontrolled hypertension were still significantly smaller compared to the observed ones among women (at -0.7%/year), and were reversed among men (at +0.38%/year).

## Discussion

To our knowledge, this is the first study that has quantified the contribution of pharmacological antihypertensive treatment to the observed decrease in the prevalence of uncontrolled hypertension in the South African adult population.

Our results suggest that the increased diffusion of treatment through the population played an important role in driving the downward trend in the prevalence of uncontrolled hypertension observed in both genders between 2008 and 2015. In the absence of treatment the decrease observed would have been substantially reduced among women and eliminated among men.

These findings are congruent with the results of a recent pooled analysis of BP data from 1018 population-based studies which examined the relative contribution of changes in mean and shape of blood pressure distribution to worldwide trends of hypertension prevalence.[45] For the sub-Saharan Africa macro-area the study concluded that the slight reduction in the age-adjusted prevalence of uncontrolled hypertension observed between 1985–1994 and 2005–2106 in men and the substantial stability of the prevalence in women happened despite increases in mean SBP and DBP. This suggests a substantial reduction in the number of individual in the right tail of the distribution, compatible with an increased diffusion/effectiveness of treatment.

This overall result, however, cannot be interpreted as a finding that the role of other factors was irrelevant. On the contrary, the comparison of age- and gender-specific observed and counterfactual trends in hypertension prevalence and mean values of SBP and DBP suggests more complex interpretations; also, that the answer to our main research question ─ that is, how much of the decline was attributable to drug therapy rather than to other factors ─ differs substantially according to age.

Among women, observed trends in mean SBP and DBP and prevalence of hypertension were consistently downward in all age categories, including in subjects 35 years or younger, among which the influence of antihypertensive treatment was negligible and counterfactual trends coincided with the observed ones. This observation implies that, at least in these age categories, factors other than treatment are driving the decrease. Conversely, among older subjects, counterfactual trends showed much smaller declines or even increases in BP, suggesting that any changes in factors other than treatment are playing a marginal or even unfavourable role.

Among men, observed trends in the prevalence of hypertension were less pronounced than among women. Also, trends in mean BP were different between SBP and DBP in some age categories. However, the findings were broadly the same as among women and that suggests that antihypertensive treatment is also a major driver of change among older male subjects, with factors other than treatment explaining the reduction among younger subjects.

Overall, these findings are congruent with the conclusions of various studies in high-income countries indicating that, even though the diffusion of effective antihypertensive treatment has undoubtedly contributed to the consistent decrease in mean BP in those populations, other factors must be necessarily involved in the reductions in the untreated, mostly younger, part of the population.[8,46,47]

The identification of these factors is, however, still an open question. The results of our adjustment for changes over time in age, BMI, waist circumference, smoking and alcohol use clearly indicate that the combined effect of these factors is not able to explain the observed trends. On the contrary, given the substantially unfavourable trends in the distribution of these factors during the study period, they suggest that even more favourable trends in BP would have been observed on the hypothesis of a stable distribution of these factors, especially BMI, in the population.

Other variables known to be strongly associated with BP and prevalence of hypertension are dietary habits, salt consumption and physical exercise, but these data were not available in the surveys analysed. However, with such a clear rise in waist circumference in both genders and in BMI among women, it would be surprising to find appreciable trends in this population towards blood pressure protective dietary habits and higher levels of physical exercise.

Early life experiences may play a role and translate in a birth cohort effect explaining the declining SBP and DBP in females and SPB in males among the younger age cohorts. The available data did not allow the direct investigation of this hypothesis, but the existence of a “favourable” birth cohort effect would be consistent with studies showing that blood pressure is decreasing in adolescents in high-income countries and possibly in some middle-income countries.[48–50] If proven, this hypothesis would portend continued declines in hypertension into the future as the younger cohorts age.

Finally, a finding which deserves further consideration is the larger absolute population effect of treatment observed among women than among men. The results of our study show that women are more frequently on treatment than men and–as indicated by the greater average treatment effects on the treated in S1 Table–they benefit more from it. An analysis of the reasons for these discrepancies is beyond the scope of our study. One likely explanation is a difference in use of or access to health services by gender. More frequent use of health care by women (for example for maternity care, contraception or cervical cancer screening, or for primary care more generally) is a well established phenomenon worldwide,[51,52] and has been repeatedly analysed in South Africa in various contexts.[53–55] Visiting health care facilities, regardless of the primary reason, increases the likelihood that asymptomatic hypertension would be detected and treated, and would also increase the effectiveness of the treatment through adherence reminders.

### Public health implications

The results of our study have direct public health implications, which are different in the various age groups.

For the older age groups, the finding that the decline in hypertension prevalence is substantially attributable to the increasing prevalence of antihypertensive therapy over time suggests that improving accessibility of treatment is an effective tool to increase hypertension control in this population. This is a positive finding, especially given the prospect of a wider diffusion of fixed-dose combination drugs. There is a growing literature that such formulations are a cost-effective alternative to current practices in many contexts, thus potentially able to favour treatment diffusion even in low-resource environments.[56,57] On the other hand, the finding that counterfactual trends in BP in the absence of treatment do not show sign of decline suggests that primary prevention efforts (e.g. public health and regulatory initiatives in place to reduce the prevalence of major risk factors for hypertension, such as obesity, malnutrition and reduced physical activity) are not producing sizable benefits in these age groups. In fact, as shown in Fig 3, net of treatment the prevalence of hypertension would be on the rise among women and stable among men.

For the younger age groups, an important finding is the decrease in the mean values of both SBP and DBP among females and in the mean SBP among males, in the absence of treatment effects and despite the simultaneous increase in waist circumference, alcohol use and BMI among females. Understanding the drivers of this seemingly paradoxical finding deserves further research efforts.

### Strengths and limitations

Strengths of the present study are the large samples including repeated measurements of blood pressure performed by trained personnel with consistent protocols and devices across the four measurement points, and the adjustment of the individual readings for seasonal variation of blood pressure. Both factors are likely to have kept to a minimum between-samples artefactual differences that could have biased trends estimates. The direct modelling of systolic and diastolic blood pressure and secondary calculation of hypertension status are also likely to have reduced the effect of the terminal digit preference and selective recording, which usually have a modest effect in mean estimation but can substantially alter estimates of the prevalence of hypertension.[10,58,59]

Unlike the ordinary methods of adjustment, in which the violation of basic statistical assumptions is known to result in biased estimates, the modelling approach used in this study for the estimation of the counterfactual values of BP and hypertension status has been shown to be a valid approach, with good performances in various contexts.[30,31,60]

Several limitations of this study need to be acknowledged.

First, the four cross-sections analysed in this study were extracted from a longitudinal study. Even though the inclusion of temporary sample members and, especially, the progressive inclusion in the successive waves of subjects who turned 15 years of age after the previous data collection resulted in a far from complete overlap between cross-sections, many individuals were present in more than one wave. This could have led to an underestimation of standard errors in trend estimates, plausibly modest, given the only partial overlap of the samples. More importantly, this could have led to an overestimation of the diffusion of treatment in the population. In fact, the protocol of the NIDS prescribed (for evident ethical reasons) that individuals whose BP measurements exceeded certain thresholds were advised to go for a doctor or hospital visit within a certain time depending on the severity of the readings. This procedure might have artificially inflated the level of awareness (and possibly the proportion of hypertensive subjects initiated on treatment) recorded in the following waves, making the sample less representative of the South African population in this regard. However, the comparison of the level of awareness and treatment in the successive waves (see S1 Fig) did not show any consistent increase from wave to wave that would support the hypothesis of an appreciable effect of the referral protocol. We consider it therefore unlikely that this phenomenon could have introduced more than a marginal overestimation of the observed downward trends in the population.

A second limitation is that a suboptimal response and greater attrition rates were observed in some social strata in the NIDS, in particular among the white subpopulation. Even though differences between respondents and non-respondents in observed characteristics have been taken into account through appropriate adjustment of sampling weights, we cannot exclude the possibility that unobserved differences might have limited the generalisability of our results to the whole South African population. In particular, given the strong association between white racial ascription and high socioeconomic status in South Africa, the applicability of our findings to high socioeconomic status strata must be considered with caution.

A third limitation is the small number of biobehavioural risk factors available in the dataset for adjustment–as mentioned above–and the low reliability of self-report data. The latter is a well known problem in population-based surveys, and this might have affected the results of our study, especially with reference to the use of antihypertensive medication, which is an important element in our modelling strategy. However, the most frequent result of low reliability in predictors of outcome variables is a bias of the observed associations towards the null,[61] and more precise measurements are therefore likely to strengthen the result of our analysis on the effect of antihypertensive treatment on BP trends rather than invalidate them.

Finally, even though the censored regression model used to estimate untreated BP requires fewer assumptions that alternative approaches (and, in particular, it does not require preliminary hypotheses regarding the magnitude of treatment effects), it is still based on a series of assumptions not directly testable with the data (see S1 File).

Among those, it assumes that, after adjustment for measured risk factors, the distribution of the true BP above any given threshold in treated subjects is the same as the corresponding distribution of observed BP in untreated subjects. This means that treated and untreated individuals only differ regarding the observed covariates, which, strictly speaking, is implausible. However, we do not believe that the likely violation of this assumption introduced major bias in our results, for three reasons. First, because the large number of predictors considered as covariates makes it less implausible that the residual differences between treated and untreated subjects after conditioning are modest if not negligible. Second, the average treatment effects in the treated individuals estimated as a weighted average of the difference between observed and counterfactual BP values (see S1 Table) were reasonable and in agreement with published meta-analyses of population studies.[62] Finally, the results of the sensitivity analysis, albeit limited, suggest that even a large amount of error in the estimation of the counterfactual values of BP is not able to change the substantive conclusions of our study.

## Conclusions

In the South African adult population, the increasing diffusion of antihypertensive treatment contributed substantially to the downward trend in the prevalence of uncontrolled hypertension and number of subjects affected observed between 2008 and 2015.

In the older age classes (55 years and older) the treatment effect also explained the observed decreases in mean SBP and DBP despite the concurrently worsening distribution of major risk factors for elevated blood pressure. Among younger subjects, a marked decrease in SBP was also present, but could not be explained either by treatment nor by the changing distribution of the other factors considered in our analyses. This requires further research.

## Supporting information

S1 Fig Proportion of subjects who reported previous diagnosis of hypertension (awareness) and proportion of subjects on antihypertensive treatment in the NIDS samples. Unweighted sample statistics.
(PDF)

S1 File Additional methods. The file provides details on the implementation of the censored regression approach for the estimation of counterfactual values of SBP and DBP.
(PDF)

S1 Table Average treatment effect on the treated (ATT) of antihypertensive medication in the South African adult population. Estimates and 95% confidence intervals.
(PDF)
