# Supplementary figures and images for: Antihypertensive treatment and blood pressure trends among South African adults: A repeated cross-sectional analysis of a population panel survey
Source: PLoS One. 2018 Aug 1;13(8):e0200606. doi: 10.1371/journal.pone.0200606 (PMC6070211; doi:10.1371/journal.pone.0200606)

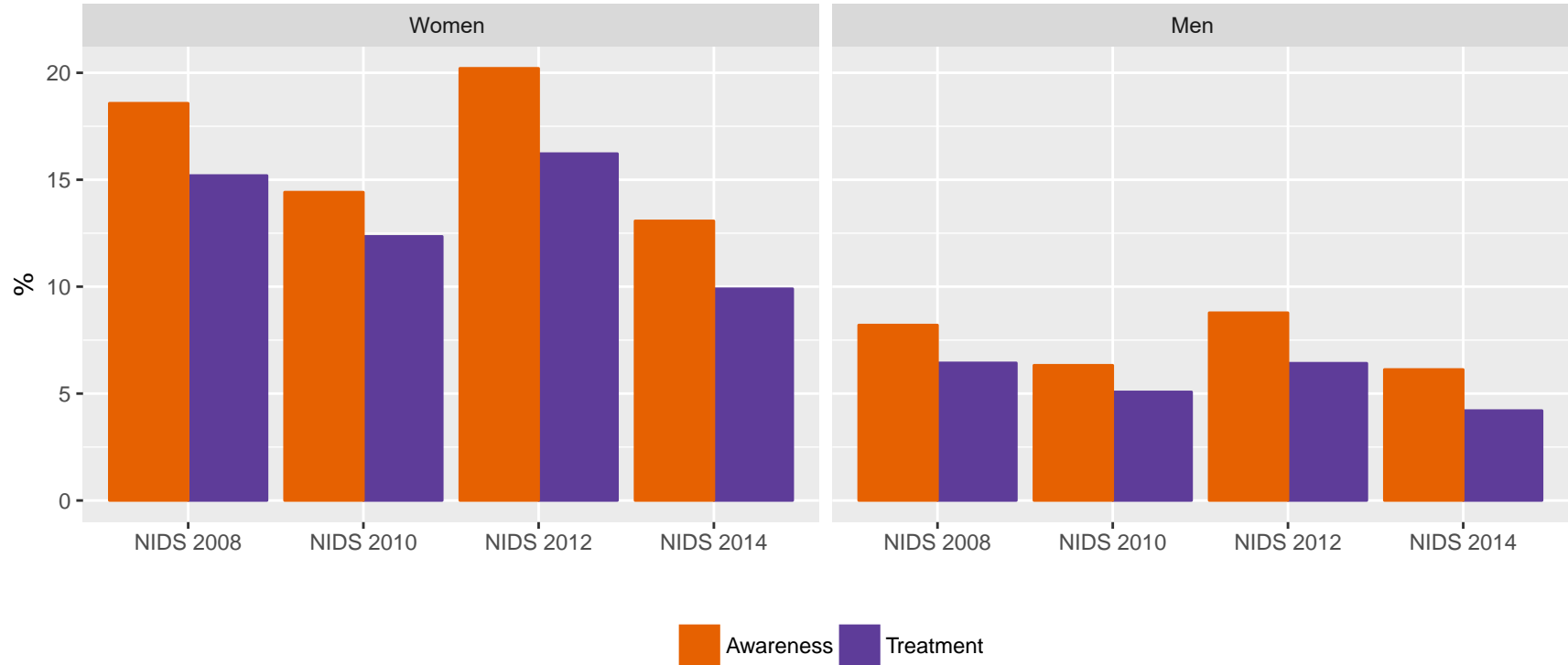

Supplement: S1 Fig — Unweighted sample statistics. (PDF) [file pone.0200606.s001.pdf]
