# Supplementary material for: Antihypertensive treatment and blood pressure trends among South African adults: A repeated cross-sectional analysis of a population panel survey
Source: PLoS One. 2018 Aug 1;13(8):e0200606. doi: 10.1371/journal.pone.0200606 (PMC6070211; doi:10.1371/journal.pone.0200606)
Supplement: S1 File — The file provides details on the implementation of the censored regression approach for the estimation of counterfactual values of SBP and DBP. (PDF) [file pone.0200606.s002.pdf]

S1 File. Additional methods

Estimation of counterfactual values of blood pressure

Separately for each SBP and DBP reading, we fitted a censored regression model with BP as the outcome and a series of demographic, biological and behavioural variables known to be associated with BP as predictors (racial ascription; age, season and their interaction; BMI; height; weight; waist circumference; current smoking and alcohol use; history of cardiovascular disease; education; urban vs. rural dwelling; province of residence).

A cosinor function and its interaction with age were also introduced among the predictors to adjust for seasonal variation as described above. We modelled the jth (j=1,2) measurement of SBP/DBP that would have been observed in absence of treatment in the ith (i=1,...n) individual in the sample as:

$$SBP_{i,j} = \beta_{0(j)} + \boldsymbol{B}_j \cdot \boldsymbol{X}_i + \epsilon_{i,j} \ , \ \epsilon_{i,j} \sim N(0, \sigma^2_{SBP(j)})$$

$$DBP_{i,j} = \beta'_{0(j)} + \boldsymbol{B}'_j \cdot \boldsymbol{X}_i + \epsilon'_{i,j} \ , \ \epsilon_{i,j} \sim N(0, \sigma^2_{DBP(j)})$$

where: SBP and DBP are considered right-censored for individuals on antihypertensive treatment (i.e. the unknown value of $SBP_{i,j}$ for treated individuals is assumed to be not lower than the observed value, in account of the plausible hypothesis that antihypertensive drugs do not increase BP) ; $\boldsymbol{X}$ is a vector of covariates including all the predictors cited above as well a series of dummy variables representing the wave of data collection; and $\boldsymbol{B}$ is a vector of regression coefficients.

We used the estimated model coefficients to predict the counterfactual BP readings of treated individuals as the conditional expectation of BP given that the observed blood pressure was lower than the true underlying BP. For SBP this translates into the formula:

$$E[SBP_{i,j}|SBP_{i,j} > SBP_{i,j}^{obs}, X_i = x_i] = \beta_{0(j)} + \boldsymbol{B}_j \cdot \boldsymbol{x}_i + \frac{\sigma_{SBP(j)} \cdot \phi(SBP_{i,j}^{obs}|x_{i,j})}{1 - \Phi(SBP_{i,j}^{obs}|x_{i,j})}$$

Where ϕ and Φ are, respectively, the normal probability and cumulative density functions with mean $\beta_{0(j)} + \boldsymbol{B}_j \cdot x_i$ and standard deviation σ_{SBPj}. An analogous formula is valid for DBP.

Assumptions

The three major assumptions underlying the applicability of this methods are: (1) the true BP of treated subjects is at least as high as the observed value under treatment (i.e., treatment does not increase BP of the treated subjects); (2) the distribution of BP in the population, conditional on the model covariates, is approximately normal; (3) the distribution of the true BP above any given threshold in treated subjects is the same as the corresponding distribution of observed BP in untreated subjects, again after conditioning on the observed covariates (uninformative censoring).

Assumption (1) and (2) are well supported by the overall epidemiological evidence regarding treatment effects and distributions of BP in large populations. The hypothesis that treated and untreated individuals only differ regarding the observed covariates – implied by assumption 3 – is, strictly speaking, unrealistic. However, the large number of covariates used for prediction in the censored regression model (which is larger than the number of covariates used for adjustment in the following steps) makes it plausible that the residual differences between treated and untreated subjects after conditioning are small, if not negligible and, therefore, we don't expect a major bias in the results of the analyses. This conclusion is supported by the substantively plausible values of the treatment effects estimated by the model and by the results of the sensitivity analysis (see Limitation section in the article).
