# Supplementary material for: Antihypertensive treatment and blood pressure trends among South African adults: A repeated cross-sectional analysis of a population panel survey
Source: PLoS One. 2018 Aug 1;13(8):e0200606. doi: 10.1371/journal.pone.0200606 (PMC6070211; doi:10.1371/journal.pone.0200606)
Supplement: S1 Table — Estimates and 95% confidence intervals. (PDF) [file pone.0200606.s003.pdf]

Period of data collection [year]	Women SBP ATT [mm Hg]	Women DBP ATT [mm Hg]	Men SBP ATT [mm Hg]	Men DBP ATT [mm Hg]
**2008**	-19.0 (-19.9;-18.0)	-12.7 (-13.3;-12.2)	-18.3 (-20.3;-16.3)	-12.1 (-13.1;-11.1)
**2010-11**	-20.0 (-21.0;-18.9)	-13.4 (-14.1;-12.7)	-18.4 (-20.3;-16.5)	-12.4 (-13.4;-11.3)
**2012**	-21.2 (-22.2;-20.4)	-14.5 (-15.1;-13.9)	-19.2 (-20.8;-17.7)	-13.1 (-14.0;-12.1)
**2014-15**	-20.7 (-21.7;-19.8)	-14.3 (-14.9;-13.6)	-19.1 (-20.6;-17.7)	-13.0 (-13.9;-12.1)
